# Case report: Novel p.Val306Met missense mutation in *TRPV3* in a case of Olmsted syndrome accompanied by squamous cell carcinoma

**DOI:** 10.3389/fonc.2024.1420555

**Published:** 2024-10-14

**Authors:** Yangyang Hao, Rong Wu, Xi Chen, Yunjia Shen, Mengwei Chou, Jianqiang Yang

**Affiliations:** ^1^ Department of Dermatology, The First Affiliated Hospital of Huzhou University, Huzhou, China; ^2^ Department of Orthopedics, The First Affiliated Hospital of Huzhou University, Huzhou, China

**Keywords:** Olmsted syndrome(OS), squamous cell carcinoma, TRPV3, missense mutation, palmoplantar keratoderma

## Abstract

Olmsted syndrome (OS) is a rare congenital skin disorder, typically characterized by symmetrical, severe palmoplantar and periorificial keratoderma, often accompanied by alopecia, and onychodystrophy, with varying degrees of pruritus and pain. Gain-of-function variants of the transient receptor potential cation channel subfamily V member 3 (*TRPV3*) were described as a cause of OS. Here, we report an atypical case of OS caused by a novel mutation in the *TRPV3* gene that has not been described before in OS. The patient presented with disabling, bilateral palmoplantar keratoderma, and subsequently developed squamous cell carcinoma on the right sole. Genetic analysis identified a novel heterozygous p.Val306Met missense mutation in the exon 8 of *TRPV3*. Our findings expand the phenotypic spectrum of *TRPV3*-related OS and underscore the need for vigilant long-term monitoring of these patients.

## 1 Introduction

Olmsted syndrome (OS, OMIM 614594) is a rare genodermatosis characterized by bilateral mutilating palmoplantar keratoderma(PPK) and periorificial keratotic plaques with severe itching and pain (1, 2). Patients may also present with alopecia, onychodystrophy, erythromelalgia, digital constriction, and pseudoainhum, with an increased risk of developing squamous cell carcinoma (SCC) and melanoma (2, 3). Studies have identified *de novo* mutations of the transient receptor potential vanilloid 3 gene (*TRPV3*) as a causative factor for OS (4). TRPV3, a thermosensitive, nonselective cation channel, is predominantly expressed in keratinocytes and sensory neurons (5). Gain-of-function mutations in *TRPV3* alter channel properties leading to enhanced keratinocyte apoptosis, hyperkeratosis, and increased epidermal turnover (4). Here, we report a Chinese case of Olmsted syndrome associated with a novel missense mutation in *TRPV3*, p.Val306Met.

## 2 Case presentation

**Figure 1:**
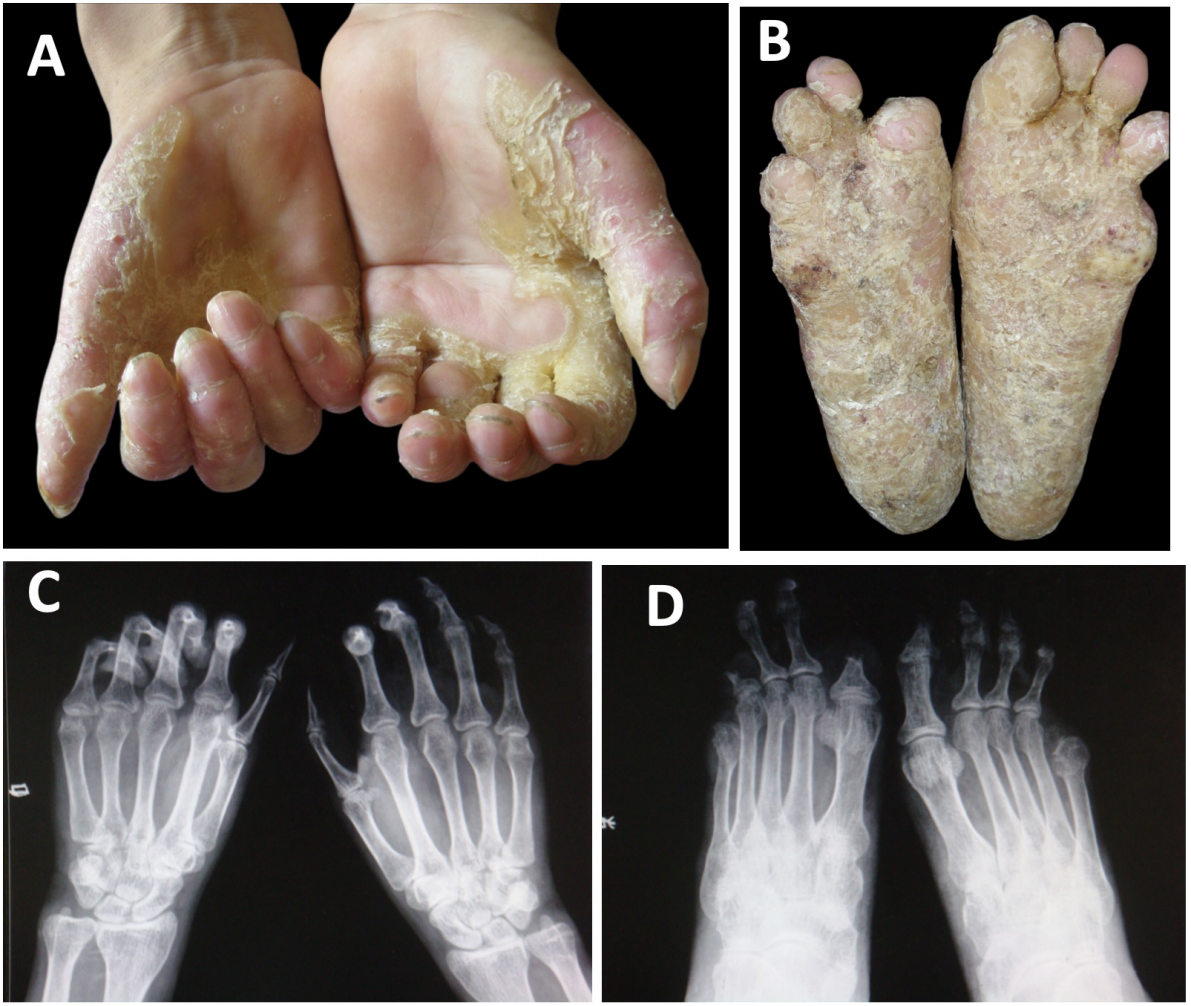
Clinical features of the patient. **(A, B)** Bilateral, mutilating palmoplantar keratoderma, flexion deformities, and constriction of digits were indicated. **(C, D)** The X-ray examination presented osteoporosis in the bones of both hands and feet, bending and deformation of phalanges. The destruction of some phalanges disappeared, and the joints were subluxated.

**Figure 2:**
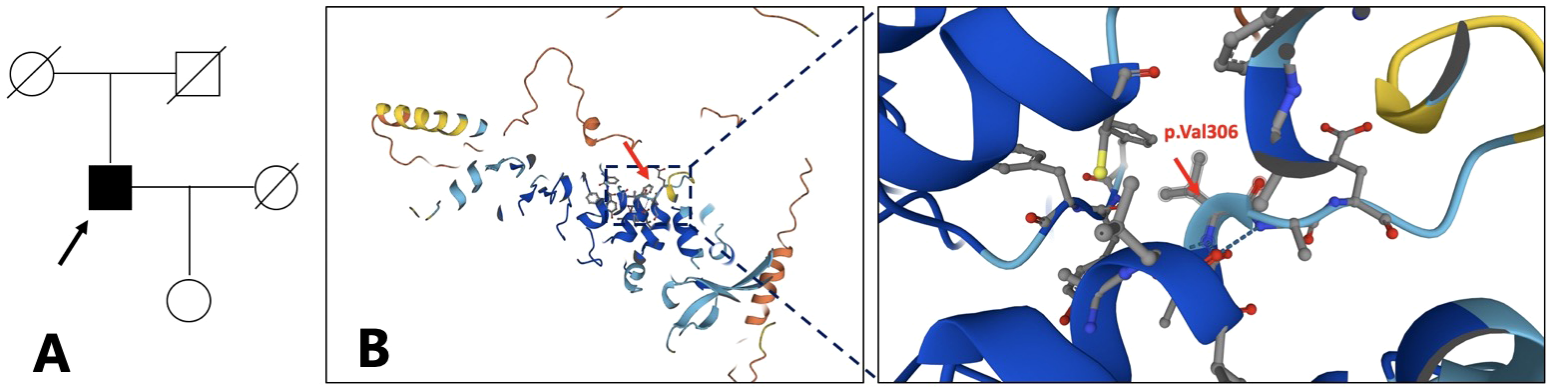
Family pedigree. The arrow refers to the proband **(A)**. The proband is 64 years old now, the proband’s father is 87 years old, the mother is 86 years old, the wife is 62 years old, and the daughter is 38 years old. Structure model of TRPV3 protein and localization of reported OS mutations **(B)**. (from https://www.genecards.org/cgi-bin/carddisp.pl?gene=TRPV3). .

A 50-year-old male patient of Chinese Han ethnicity presented to our department with severe bilateral palmoplantar hyperkeratosis, accompanied by intense pain and itching, persisting for more than 40 years. The patient exhibited thick, sharply marginated, yellowish hyperkeratotic plaques with a foul odor on both palms and soles leading to flexion contractures of the digits that severely limited his mobility. The phalangeal joints appeared thinned and annularly narrowed, ultimately resulting in spontaneous amputations. Notably, the first, fourth, and fifth toes of the right foot and the fifth toe of the left foot were absent, with the remaining toes showing varying degrees of contracture deformity (**Figures 1A, B**). No abnormalities were observed on the skin of the trunk and limbs, and the patient’s hair, teeth, and hearing were normal. His medical history was unremarkable for other diseases such as diabetes, hypertension, or vascular disease. His parents were healthy and non-consanguineous, and no similar symptoms were reported in his daughter (**Figure 2A**).

Routine blood tests revealed mild anemia, while routine chemistry panels showed no abnormalities. The patient’s blood sugar level is normal (fasting blood glucose 5.1 mmol/L, 2-h postprandial blood glucose 7.2 mmol/L). X-ray examination of the hands and feet revealed osteoporosis, bending and deformation of the phalanges, and subluxation of joints. In some areas, phalangeal destruction was noted, with complete disappearance of certain bones (**Figures 1C, D**).

**Figure 3:**
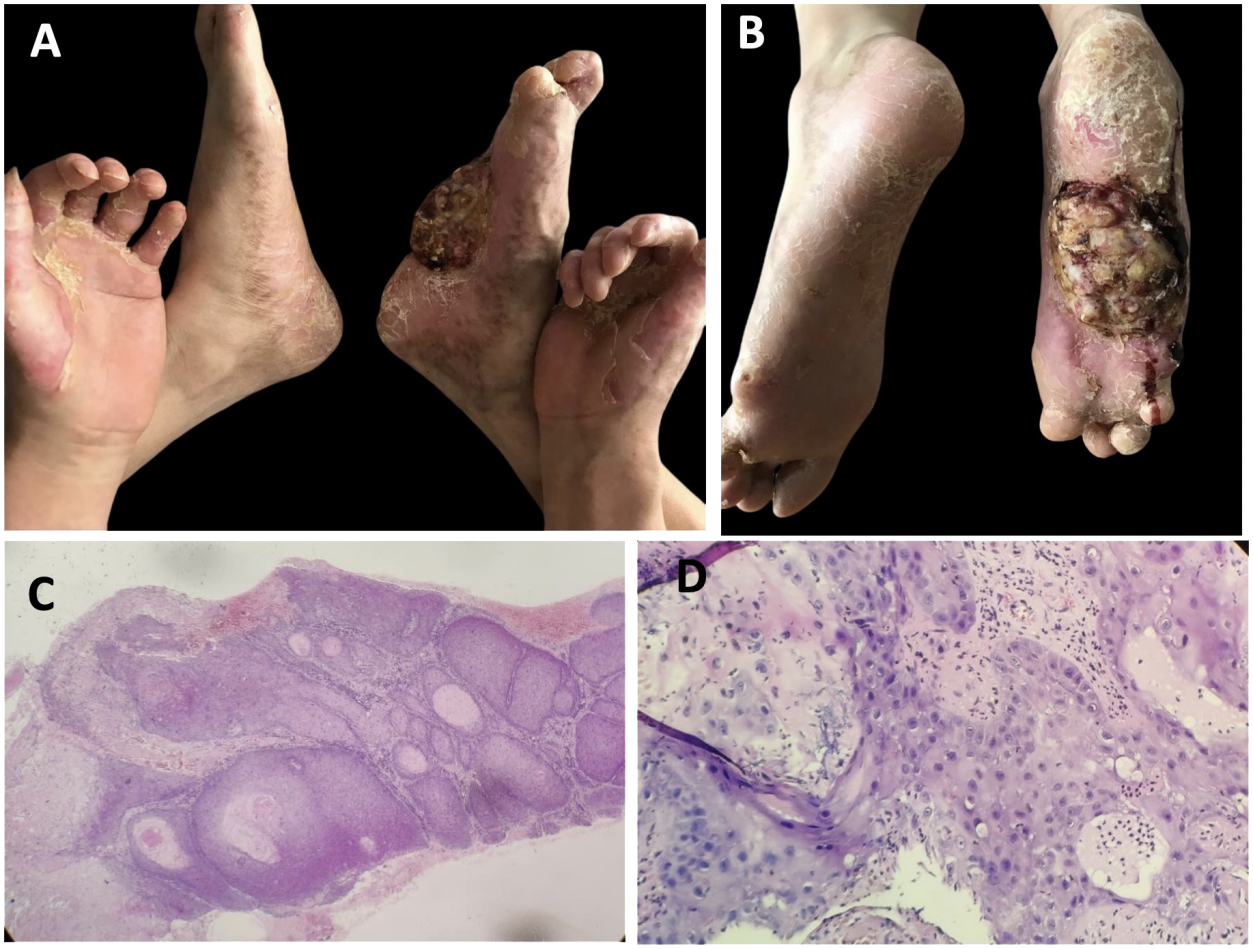
Clinical features and pathological characteristics of the patient followed-up 5 years later. **(A, B)** The tumor was present on the sole of the right foot, accompanied by bleeding and ulcer. **(C, D)** Hyperkeratosis, marked epidermal thickening, with cellular and nuclear heterogeneity and atypia, and abnormal mitotic activity, which indicated squamous cell carcinoma. (hematoxylin–eosin, ×100 and ×400, respectively).

The patient experienced moderate improvement following a 6-month treatment with systemic acitretin (10 mg, three times a day), topical keratolytics (0.1% Tretinoin cream, twice a day), and moisturizers, though treatment was administered intermittently over many years. No significant side effects were observed during the treatment period. Five years later, a lump appeared on the right sole, which gradually enlarged and was accompanied by bleeding and ulceration (**Figures 3A, B**). Histological examination of a biopsy confirmed the diagnosis of well-differentiated squamous carcinoma (**Figures 3C, D**). Due to the size of the tumor and invasive nature, the patient underwent amputation of his right foot to prevent further spread and save his life.

**Figure 4:**
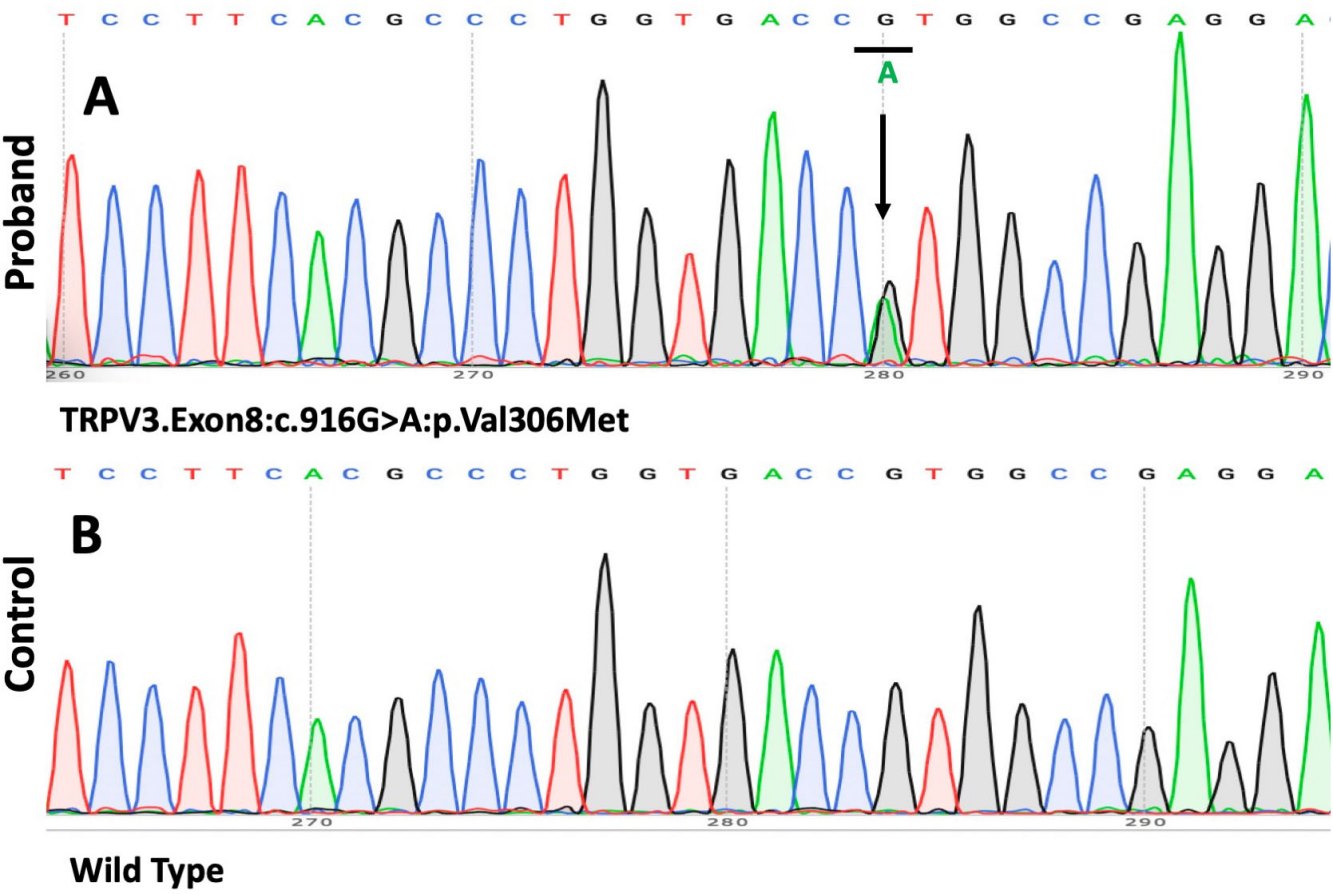
The results of the mutation analysis. **(A, B)** A heterozygous 916G>A mutation (p.Val306Met) was found in the gene *TRPV3* in the patient.

With informed consent, genomic DNA was extracted from the peripheral blood of the proband and his daughter, as the proband’s parents were deceased. Whole-exome sequencing (WES) and Sanger sequencing of the genomic DNA identified a novel heterozygous G>A transition at position c.916 in exon 8 of *TRPV3* leading to the substitution of a valine residue to methionine (p.Val306Met). This mutation, which has not been previously described in OS, was absent in the patient’s daughter and healthy controls. We did not detect any pathogenic sequence alterations in palmoplantar hyperkeratosis-related genes such as *KRT1*, *GJB2*, *LOR*, *SLURP1*, *MBTPS2*, *PERP*, or *CTSC* (**Figures 4A, B**).

ConSurf analysis demonstrated that this region forms a highly conserved section of the channel pore of TRPV3. Multiple bioinformatics tools, including SIFT, PolyPhen-2, and MutationTaster, were used to assess the pathogenicity of this variant. SIFT and MutationTaster predict the p.Val306Met variant to be pathogenic (SIFT:0;MutationTaster:1), while Polyphen2 suggests the variant is likely benign(Polyphen2:0.322).

## 3 Discussion

The transient receptor potential vanilloid 3 (TRPV3) is a thermosensitive, nonselective cation channel that plays a significant role in mediating itch and pain sensation, regulating keratinocyte proliferation and differentiation, hair growth, and inflammation (6) (**Figure 2B**). Electrophysiological experiments have demonstrated that gain-of-function mutations in *TRPV3* can exert significant pathogenic effects on protein function, leading to the characteristic pathological phenotype of Olmsted syndrome (OS) (7). Additionally, mutations in the membrane-bound transcription factor protease, site 2 (*MBTPS2*) gene, and the *PERP* gene have been identified as other causes of OS (8, 9). Clinically, OS must be differentiated from other conditions like Vohwinkel syndrome and Papillon–Lefèvre syndrome, both of which present with overlapping symptoms but are caused by different genetic mutations.

The TRPV3 channel is essential for maintaining normal physiological functions. The pathogenesis of OS remains unclear, but it is hypothesized that TRPV3 gain-of-function mutations lead to increased intracellular calcium levels, inducing keratinocyte apoptosis, and contributing to the characteristic hyperkeratosis of OS (4). Recent studies have also suggested that *TRPV3* plays a role in lysosomal function, and mutations in this gene may contribute to lysosomal dysfunction, further complicating the clinical presentation of OS. *TRPV3* has been detected in the lysosome, and studies have shown that the p.Gly568Cys and p.Gly568Asp mutants in *TRPV3* induce a decrease in lysosomal numbers, less lysosomal movement, and less acidification of lysosomes. The p.Gly568Cys and p.Gly568Asp mutants differ in lysosomal pH regulation and movement. These differences in lysosomal properties may be related to different OS phenotypes (10). OS mutants also cause reduced cell adhesion, altered distribution, and reduced number of lysosomes, which may have a broad implication for the keratinocyte functions, skin degeneration, and skin cancer (11). Additionally, TRPV3 is endogenously expressed in macrophages, and activation of TRPV3 can efficiently eliminate bacteria (12).

A review of reported OS cases highlight the heterogeneity of its clinical phenotype. Typical manifestations of OS include diffuse palmoplantar keratosis and periorificial keratotic plaques, whereas atypical symptoms of OS may involve limited or absent periorificial keratotic plaques. To date, at least 21 *TRPV3* gene variants have been reported in OS patients, with some mutations, such as p.Gly573Cys and p.Trp692Gly, being more common and typically associated with classic OS presentations (2, 13). Mutations at other positions tend to be linked to atypical presentations of the syndrome (7). The clinical manifestations of our patient were atypical, as only palmoplantar hyperkeratosis was observed, with no periorificial keratotic plaques or other OS-related symptoms. The p.Val306Met mutation in *TRPV3* of our patient is a novel heterozygous missense mutation. However, ConSurf analysis demonstrated that this region forms a highly conserved section of the channel pore of TRPV3. Two silico tools predict the p.Val306Met mutation to be pathogenic (SIFT: 0; MutationTaster: 1), while Polyphen2 suggests the variant is likely benign (Polyphen2:0.322). The discrepancies in these predictions highlight the complexity of assessing this variant’s pathogenicity based solely on *in silico* analysis. We did not detect any pathogenic sequence alterations in palmoplantar hyperkeratosis-related genes such as *KRT1*, *GJB2*, *LOR*, *SLURP1*, *MBTPS2*, *PERP*, or *CTSC.* Thus, we consider that the patient is likely an atypical OS caused by p.Val306Met mutation in *TPRV3*. While bioinformatics predictions provide valuable insights, they are not definitive. To conclusively determine the pathogenicity of the p.Val306Met variant, further experimental studies are essential. These studies would ideally include functional assays to assess the impact of the variant on TRPV3 protein function and clinical correlation studies in affected individuals.

A significant diversity in clinical manifestations has been reported in *TRPV3*-related OS cases, and there is currently no consensus on the genotype–phenotype correlation. However, Zhong et al. have made a preliminary investigation into this relationship (7). *TRPV3* contains six transmembrane domains (S1–S6), and mutations in the S4–S5 linker and the TRP domain of *TRPV3* have a profound impact on channel properties leading to severe clinical manifestations. In contrast, mutations in other domains tend to affect the channel properties slightly resulting in milder forms of OS. The 3D structure model of TRPV3 protein referenced from the Genecards website, which is a predicted model obtained from AlphaFold, shows the position of p.Val306 (**Figure 2B**). The p.Val306Met mutation in our patient, which in a way forms the S4–S5 linker and the TRP domain of *TRPV3*, may have a lesser impact on the channel function potentially leading to milder and atypical OS. Further electrophysiological experiments are required to verify the effect of the p.Val306Met mutation on channel properties. We suspect that p.Val306Met mutation may cause milder and atypical OS, but as OS is a progressive disease, clinical symptoms may worsen over time leading to more severe disability.

Notably, the patient developed squamous cell carcinoma (SCC) at the site of the lesion several years after the initial diagnosis, which is relatively rare. The probability of SCC occurring in OS is relatively low. Among the currently reported OS cases, two cases have developed SCC. A 48-year-old woman who developed several squamous cell carcinomas of the limbs and adenocarcinoma of the lung was diagnosed with OS (14). Another OS patient developed SCC on his right foot. Chronic inflammation, recurrent infections, and mechanical stimulation by hyperkeratosis may contribute to the development of SCC in OS patients (15). The changes in ion channel function and lysosomal dysfunction caused by *TRPV3* gene mutations may be among the reasons why OS patients develop SCC.

Currently, there are still no effective therapies for OS. Topical treatments, including keratolytics, retinoids, corticosteroids and emollients, as well as systemic therapies, such as retinoids and methotrexate, can alleviate symptoms but have limited efficacy (16). 17(R)-resolvin D1, a fatty acid-derived resolvin, which can specifically inhibit *TRPV3*-mediated activity, and low-dose epidermal growth factor receptor inhibitor, erlotinib, may be the promising future treatments for OS (17, 18). Our patient experienced symptom relief with low-dose oral acitretin, topical moisturizers, and retinoids. However, due to the development of SCC, his right foot was amputated as the tumor could not be removed. Clinicians should remain vigilant for the potential development of SCC on keratotic lesions, and early intervention may be beneficial for patients.

## Data Availability

The original contributions presented in the study are included in the article material, further inquiries can be directed to the corresponding author.
